# Infected Right Ventricle Thrombus as a Cause of Persistent Sepsis

**DOI:** 10.7759/cureus.10751

**Published:** 2020-10-01

**Authors:** Antony J Arumairaj, Noella Boma, Savi Mushiyev, Morcos Morcos, Imnett Habtes

**Affiliations:** 1 Internal Medicine, Metropolitan Hospital Center, New York, USA; 2 Cardiology, Metropolitan Hospital Center, New York, USA; 3 Radiology, Metropolitan Hospital Center, New York, USA; 4 Pulmonary and Critical Care Medicine, Metropolitan Hospital Center, New York, USA

**Keywords:** infected cardiac thrombus, sepsis, mssa bacteremia, intravenous drug user, cocaine, surgical resection

## Abstract

The presentation of fevers in a patient with active intravenous (IV) drug use is often challenging, as there is a wide range of both infectious and noninfectious disorders that can cause fevers. A thorough diagnostic workup is essential in identifying the etiology of these fevers. We report a rare case of an infected right ventricular (RV) thrombus as a cause of persistent fever and sepsis in a 46-year-old patient with IV drug use. The patient continued to have persistent bacteremia inspite of appropriate IV antibiotics. Hence, the patient warranted a cardiothoracic surgical excision of the infected RV thrombus following which the patient showed remarkable improvement.

## Introduction

Chronic intravenous (IV) drug users are predisposed to develop sepsis and bacteremia. However, an infected cardiac thrombus as a cause of persistent sepsis in a patient with IV drug use is a rare phenomenon. The development of cardiac thrombus and subsequent infection of the thrombus indicates that there has been a chronic insult to the endocardium from the unsterile IV drug use [1]. The complications from dissemination and seeding of the thrombus can lead to significant morbidity and mortality [2]. Hence, the early diagnosis of the infected cardiac thrombus with appropriate medical and surgical management can prevent life-threatening complications. We report a rare case of infected right ventricular (RV) thrombus as a cause of persistent fever and sepsis in a patient with chronic IV drug use.

## Case presentation

A 46-year-old man with a background history of active IV drug use and chronic alcohol consumption presented to the emergency department with persistent fevers, epigastric pain, nausea, vomiting, diffuse myalgia, and dysuria for the past week. The patient reported drinking four liters of alcohol per day for a week before the presentation and reported injecting IV cocaine daily until the day before the presentation. The patient was noted to have multiple injection marks in the upper and lower extremities.

**Figure 1:**
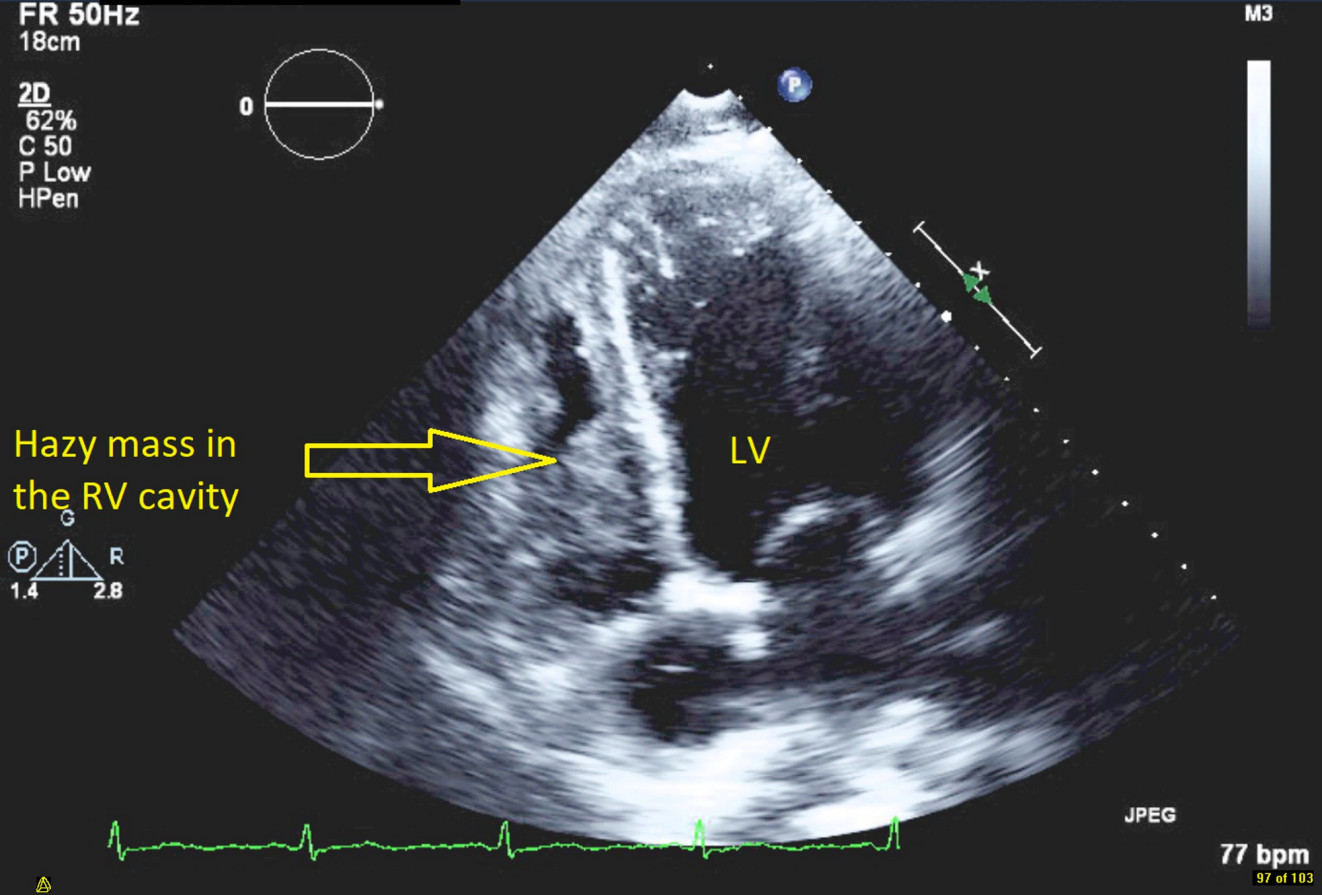
Transthoracic echocardiogram apical four-chamber view showing hazy density in the cavity of the right ventricle (RV) suggestive of a mass along with left ventricle (LV).

**Figure 2:**
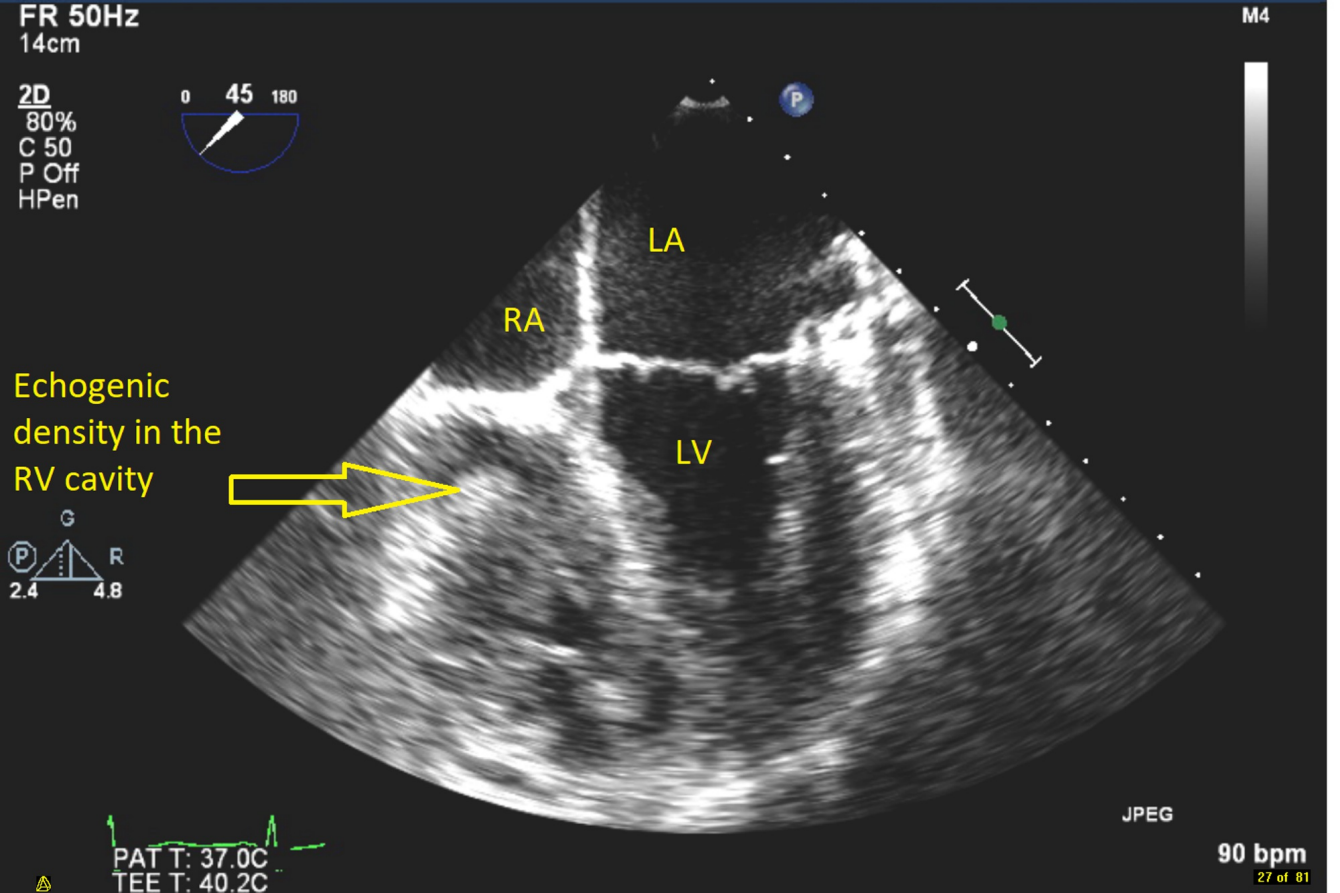
Transesophageal echocardiogram mid esophageal view showing a clearer echogenic density in the cavity of the right ventricle (RV) measuring 31 x 20 mm suggestive of a mass along with right atrium (RA), left ventricle (LV), and left atrium (LA).

The temperature on arrival was 103.1 degrees Fahrenheit with a heart rate of 99 beats/minute. Initial labs showed a normal white blood cell count, hemoglobin, and lactic acid level. The initial chest x-ray (CXR) did not show any pulmonary infiltrates. The electrocardiogram showed normal sinus rhythm. Cultures were drawn, and the patient was started on broad-spectrum IV antibiotics with vancomycin and cefepime. CT of the abdomen showed diffuse thickening of the bladder wall likely representing cystitis and incidentally showed a filling defect in the RV thought to represent a thrombus. Transthoracic echocardiogram (TTE) showed a hazy density in the cavity of the RV with normal left ventricular ejection fraction (Figure 1). Subsequently, the patient underwent a transesophageal echocardiogram (TEE) with contrast that showed an RV mass measuring 31 x 20 millimeters (mm) (Figure 2). The patient continued to have recurrent fevers, and serial blood cultures were collected. Venous duplex of bilateral upper extremities showed no evidence of deep vein thrombosis. During the hospital stay, the patient developed sudden onset of right-sided pleuritic pain. Repeat CXR showed right lower lobe consolidation with pleural effusion. Blood cultures and the urine cultures grew methicillin sensitive Staphylococcus aureus (MSSA), and IV antibiotics were deescalated to cefazolin. The patient however continued to have low-grade fevers despite tailored antibiotics and was therefore transferred to a tertiary care center for evaluation and management by the cardiothoracic surgery.

**Figure 3:**
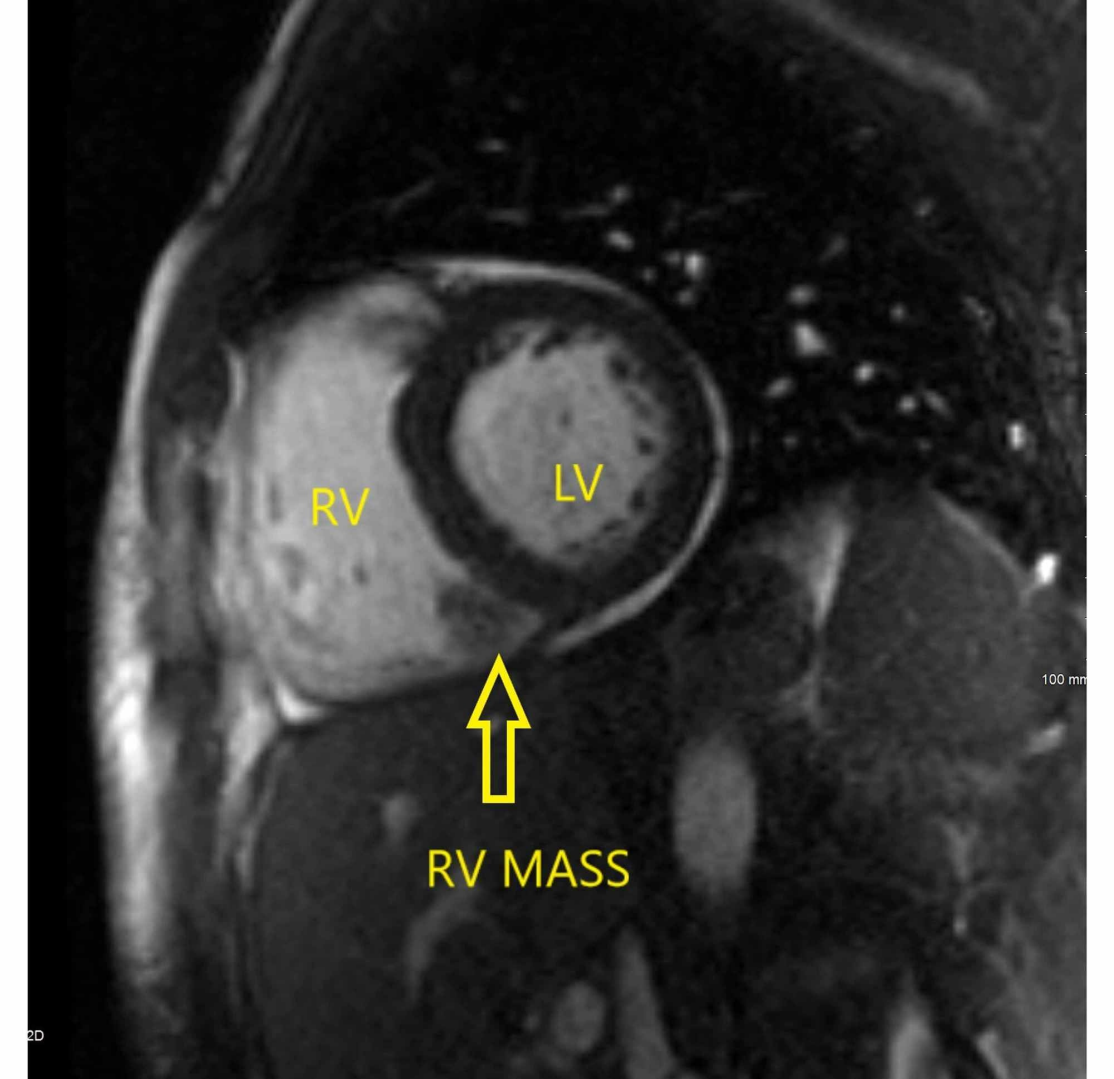
Bright blood two-chamber view image from a cardiac magnetic resonance study demonstrates a mass that appears attached to the inferior wall of the right ventricle (RV mass) measuring 30 x 17 x 23 mm along with the left ventricle (LV).

**Figure 4:**
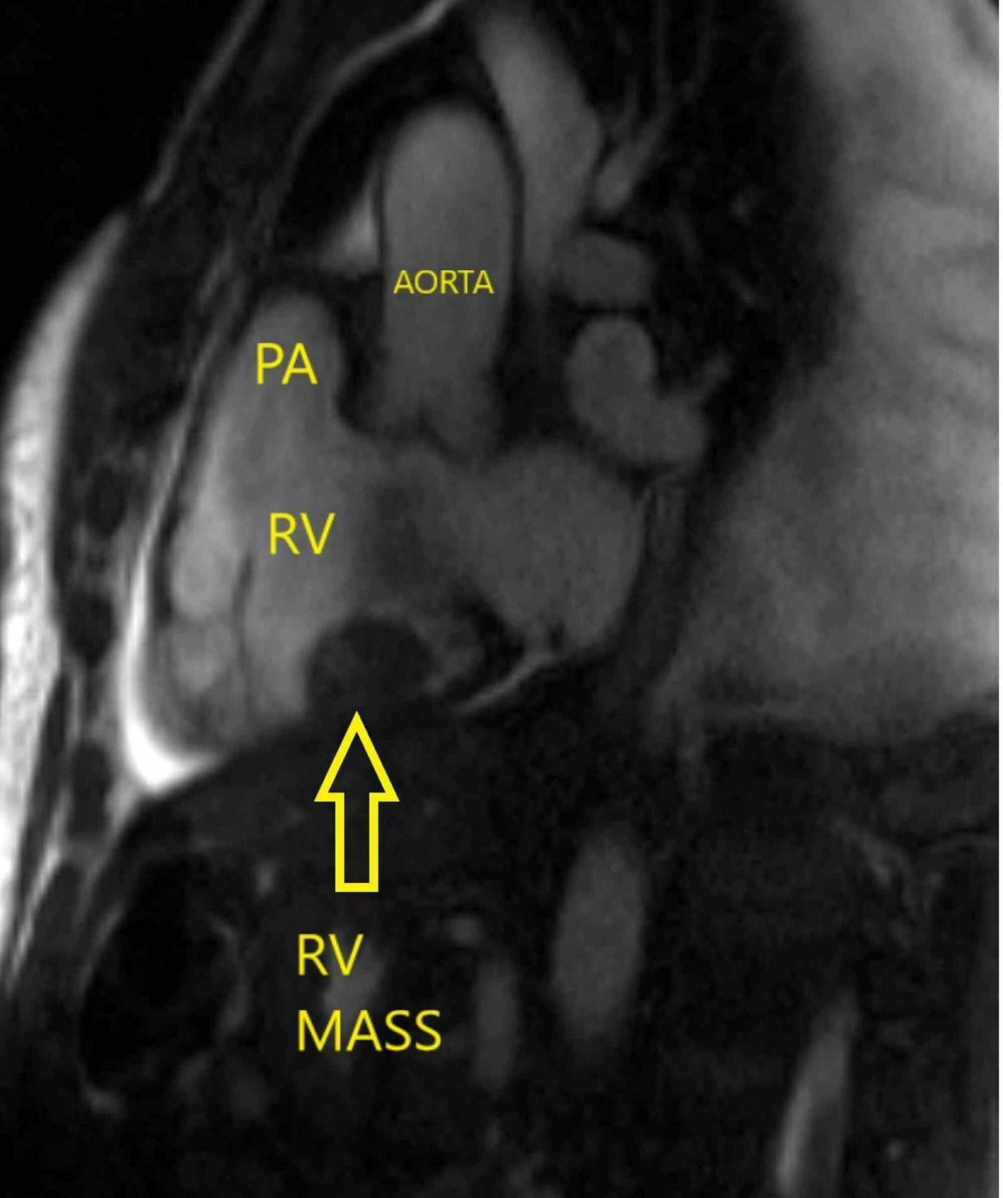
Bright blood outflow tract image from a cardiac magnetic resonance study demonstrates a mass attached to the inferior wall of the right ventricle (RV mass) measuring 30 x 17 x 23 mm along with the pulmonary artery (PA) and aorta.

After transfer, the patient underwent cardiac magnetic resonance imaging (MRI) that showed RV mass measuring 30 x 17 x 23 mm adherent to the RV and attached to the septal tricuspid papillary muscle creating severe tricuspid regurgitation (TR) (Figures 3, 4). There was also a small lesion measuring 10 mm adherent to the RV apex which possibly represented a small thrombus. There was no obvious vegetation seen. The antibiotics were changed from cefazolin to nafcillin, and a peripherally inserted central catheter (PICC) was placed for a prolonged treatment course. MRI brain and CT cerebral angiogram were normal. The patient was started on therapeutic anticoagulation with enoxaparin. The patient continued to have low-grade fevers. Serial blood cultures continued to show the growth of MSSA. In a patient with an RV thrombus, the persistence of bacteremia and sepsis despite appropriate antibiotics indicates the failure of medical management and should raise suspicion for an infected RV thrombus [3,4]. The source of sepsis had to be removed. Hence, the patient warranted a surgical intervention for removal of the thrombus [5].

The patient underwent cardiothoracic surgical excision of the RV mass and the smaller apical mass. The chordae supporting the septal leaf of the tricuspid valve were ruptured that resulted in severe TR. These chordae were repaired, and the tricuspid valve was noted to be free of any vegetation. The patient had an uneventful postoperative recovery. The excised masses were sent for culture, gram stain, and pathologic analysis. The RV mass tissue culture showed no growth, and the gram stain was negative. The pathology reports showed fibrinous thrombus with acute inflammation and focal organization. The patient improved symptomatically with complete resolution of fevers. The course of Nafcillin was completed after six weeks. Repeat postoperative TTE showed no RV thrombus or TR. The patient was discharged to an inpatient rehabilitation facility. During his inpatient rehabilitation, the patient continued to show symptomatic improvement and recovered to his baseline functional status. The patient was eventually discharged back to his home.

## Discussion

The first step in the formation of the RV thrombus is the endocardial injury caused by contaminating debris from IV drug injection. The endothelial damage triggers a sterile thrombus formation, which occurs by the deposition of fibrin and platelets [6]. In addition, the patient’s active IV drug use, particularly of cocaine, with its procoagulant properties predisposed to the formation of an RV thrombus [7]. The penetration of peripheral veins in an unsterile manner led to bacteremia causing the RV thrombus to become infected [8]. Once bacteria attach to the endocardium, the thrombus matures through additional deposition of fibrin and bacterial proliferation and the bacteria seeds under the surface of the thrombus. The infected RV thrombus became the cause of persistent sepsis by hematogenous dissemination into the pulmonary and systemic circulation causing pneumonia and cystitis [9].

TEE is essential in the diagnosis of intracardiac thrombus. As seen by our patient, TTE has been found to have a lower sensitivity for the detection of vegetations (40%-60%) as compared to TEE (94%-100%) [10]. Cardiac MRI is also advantageous because it can provide excellent delineation of anatomic structures using their physical and biochemical properties. The ability to obtain images in multiple planes adds to its versatility and diagnostic utility and offers special advantages for surgical treatment and planning [11].

Surgical resection followed by a prolonged course of antibiotics is the definitive management of a large infected cardiac thrombus [12]. However, patients need to have a good ventricular function in areas other than the site of the thrombus to withstand surgical intervention. In patients who have a poor cardiopulmonary reserve, the ventricular function should be optimized before proceeding with surgery. Medical management with a prolonged course of antibiotics has been reported as a last resort for patients who have unstable hemodynamics and have a very high surgical risk [13]. There is no standard treatment protocol for patients with an infected cardiac thrombus; therefore, each patient requires an individual treatment plan based on the patient's comorbid conditions and the size of the thrombus [14].

## Conclusions

A high index of clinical suspicion is essential in early recognition of an infected cardiac thrombus. Surgical resection of the infected cardiac thrombus remains the definitive treatment. Following the removal of the source of sepsis, a prolonged course of IV antibiotics is mandatory for the complete resolution of sepsis. Instituting appropriate medical management followed by timely surgical intervention in our patient resulted in remarkable improvement before the potential embolic dissemination of the infected cardiac thrombus and lead to an uneventful recovery.
